# Устойчивость к возбудителям фитофтороза и глободероза современного сортимента семенного картофеля и его фитосанитарное состояние в различных агроклиматических зонах европейской части России

**DOI:** 10.18699/VJ20.629

**Published:** 2020-07

**Authors:** A.V. Khiutti, D.A. Rybakov, T.A. Gavrilenko, O.S. Afanasenko

**Affiliations:** All-Russian Institute of Plant Protection, Pushkin, St. Petersburg, Russia Institute of Cytology and Genetics of Siberian Branch of the Russian Academy of Sciences, Novosibirsk, Russia; Federal Research Center the N.I. Vavilov All-Russian Institute of Plant Genetic Resources (VIR), St. Petersburg, Russia; Federal Research Center the N.I. Vavilov All-Russian Institute of Plant Genetic Resources (VIR), St. Petersburg, Russia Institute of Cytology and Genetics of Siberian Branch of the Russian Academy of Sciences, Novosibirsk, Russia; All-Russian Institute of Plant Protection, Pushkin, St. Petersburg, Russia Institute of Cytology and Genetics of Siberian Branch of the Russian Academy of Sciences, Novosibirsk, Russia

**Keywords:** distribution of potato diseases, Phytophthora infestans, Globodera rostochiensis, potato cultivars, resistance to diseases, Helminthosporium solani, Colletotrichum coccodes, DNA markers., распространенность болезней картофеля, Phytophthora infestans, Globodera rostochiensis, серебристая парша, антракноз, сорта картофеля, устойчивость, ДНК-маркеры.

## Abstract

Активная экспансия зарубежных сортов картофеля на территорию Российской Федерации привела к смене доминирующих видов патогенов этой культуры и появлению новых патотипов возбудителей вредоносных болезней. Целью работы была оценка устойчивости к возбудителям фитофтороза и глободероза современного сортимента картофеля и определение поражаемости возделываемых сортов картофеля грибными и оомицетными болезнями в различных агроклиматических зонах России. Проведена оценка устойчивости 41 сорта зарубежной селекции, разрешенного к использованию на территории РФ, к патотипу Ro1 Globodera rostochiensis и к изоляту VZR17 Phytophthora infestans, включающего гены вирулентности 1.2.3.4.5.6.7.8.9.10.11. Устойчивыми к золотистой картофельной нематоде оказались 38 сортов. У 96.6 % изученных нематодоустойчивых сортов выявлен маркер гена Н1 устойчивости к патотипу Ro1 G. rostochiensis, восприимчивые сорта этим маркером не обладали. Абсолютной устойчивостью к возбудителю фитофтороза отличались сорта Alouette и Sarpo Mira (балл 9); высоким уровнем устойчивости (баллы 6 и 7) характеризовались сорта Evolution, Red Fantasy и Ricarda. Сорта Baltic Rose, Damaris, Desiree, Gala, Labella, Laperla, Mia, Sanibel, Zekura, Queen Anne, Red Lady и 7 for 7 были отнесены к восприимчивым, хотя в характеристиках оригинаторов указана средняя устойчивость к фитофторозу. Фитопатологическая экспертиза проведена для 92 образцов 39 сортов семенного картофеля из четырех федеральных округов РФ: Приволжского, Северо-Западного, Центрального и Северо-Кавказского. Наибольшее распространение на всех сортах получили ризоктониоз, сухая фузариозная гниль и серебристая парша. Стопроцентное поражение клубней серебристой паршой отмечено в различных регионах на сортах элитных репродукций Red Scarlett, Evolution, Labella, Colombo, Gala, Невский. Широко распространен антракноз картофеля; сильнее всего были поражены клубни элитной и второй репродукции сорта Red Scarlett – от 50.0 до 71.4 % в Центральном федеральном округе.

## Abstract (en)

The active expansion of foreign potato cultivars on the territory of the Russian Federation has led to a change in the dominant pathogen species and to the emergence of new pathotypes of causal agents of harmful potato diseases. The aim of the study was to evaluate resistance to Phytophthora infestans and Globodera rostochiensis of modern potato cultivars and determine the distribution of fungal and oomycetic diseases on potato cultivars in various agroclimatic zones of Russia. The resistance of 41 foreign cultivars was evaluated to pathotype Ro1 G. rostochiensis and to isolate VZR17 P. infestans with virulence genes 1.2.3.4.5.6.7.8.9.10.11. Resistant to G. rostochiensis were 38 cultivars. 57R marker of the H1 gene conferring resistance to the Ro1 pathotype of G. rostochiensis was detected in 96.6 % of the nematode resistant cultivars studied; susceptible varieties did not possess this marker. Absolute resistance to the causative agent of late blight was demonstrated by the cultivars Alouette and Sarpo Mira (score 9); high levels of resistance (score 6 and 7) were determined for the cultivars Evolution, Red Fantasy and Ricarda. The cultivars Baltic Rose, Damaris, Desiree, Gala, Labella, Laperla, Mia, Sanibel, Zekura, Queen Anne, Red Lady and ‘7 for 7’ were classified as susceptible, although the characteristics of originators indicated average resistance to late blight. A phytopathological test was conducted on 92 samples of 39 varieties of seed potatoes from four federal districts of the Russian Federation: Volga, NorthWest, Central and North Caucasus. Rhizoctonia solani, Fusarium spp. and Helminthosporium solani are most common on all varieties. 100 % defeat of tubers by H. solani was recorded in various regions on the cultivars Red Scarlett, Evolution, Labella, Colombo, Gala and Nevsky. Widespread Colletotrichum coccodes on tubers of the elite and 2nd reproductions of the potato cultivar Red Scarlett (50.0–71.4 %) was recorded in the Central District

## Introduction

В 2015 г. в Берлине, на Международном конгрессе по защите растений (IPPC 2015) были представлены данные о том, что на картофеле даже при применении химических средств защиты потери от болезней составляют 25–30 % (Oerke, 2006).

Российская Федерация является лидером по выращиванию зарубежных сортов картофеля: из 455 сортов, включенных в «Государственный реестр селекционных достижений, допущенных к использованию» в 2019 г., 182 (40.0 %) – селекции стран дальнего зарубежья, 34 (7.5 %) – стран СНГ, и только 239 (52.5 %) сортов созданы российскими селекционерами (www.gossortrf.ru). Отметим также, что в большинстве картофелеводческих хозяйств представленность зарубежных сортов достигает 100 %.

Активная экспансия зарубежных сортов и несоблюдение регламента по их агротехнике привели к плачевному результату: на территории РФ распространились новые патотипы, относящиеся как к оомицетам и грибам, так и к вирусам, бактериям и нематодам (Еланский, 2015; Кузнецова и др., 2016). Кроме того, появляются новые патогены, например возбудители черной ножки и мокрой гнили Pectobacterium carotovorum subsp. brasiliense, P. carotovorum subsp. odoriferum, P. parmentieri, новый для России вирус P (PVP), возбудитель розового фитофтороза Phytophthora erythroseptica Pethybr. (Игнатов и др., 2019; Yanagisawa et al., 2019).

Наиболее вредоносными в России являются следующие оомицетные и грибные болезни картофеля: фитофтороз (Phytophthora infestans (Mont.) de Bary), потери урожая от которого без применения химических средств защиты варьируют от 80 до 100 %; комплекс паршей картофеля, включающий ризоктониоз (Rhizoctonia solani Kühn), серебристую (Helminthosporium solani Durieu et Mont.), обыкновенную (Streptomyces spp.), бугорчатую (Polyscytalum pustulans (Owen & Wakef.) Ellis) и порошистую (Spongospora subterranea f. sp. subterranea Toml.) паршу, потери от которых достигают 30 %; а также антракноз (Colletotrichum coccodes (Wallr.) Hughes) – потери до 20-30 %, сухая фузариозная гниль (грибы рода Fusarium spp.) и фомоз (Phoma spp.) – потери не менее 20 %, и объект как внутреннего, так и внешнего карантина – золотистая картофельная нематода (Globodera rostochiensis (Wollenweber, 1923) Skarbilovich, 1959), вредоносность которой достигает 80–90 % (Winslow, Willis, 1972; Dillard, 1992; Johnson, Miliczky, 1993; Johnson, 1994; Tsror et al., 1999; Collins, 2000; Lees, Hilton, 2003; Judelson, Blanco, 2005; Haldar et al., 2006; Gudmestad et al., 2007; Haverkort et al., 2009; Tsror, 2010; Abbas et al., 2013).

Конкурентоспособность сортов картофеля определяется главным образом устойчивостью к наиболее вредоносным в зоне возделывания болезням. В связи с этим создание сортов картофеля, устойчивых к основным болезням, является приоритетным направлением селекции. Доля устойчивых к болезням сортов картофеля, зарегистрированных в Госреестре селекционных достижений, с каждым годом увеличивается. Наиболее существенные результаты получены при селекции картофеля на устойчивость к карантинным болезням. Все новые сорта, внесенные в Госреестр, отличаются устойчивостью к возбудителю рака картофеля, кроме четырех старых сортов (Волжанин, Ермак улучшенный, Лорх и Приобский), доля которых составляет 0.6 %. К настоящему времени 55.4 % сортов, включенных в Госреестр, устойчивы к золотистой картофельной нематоде (ЗКН) (www.gossortrf.ru). Зарегистрированные в Госреестре в 2019 г. 22 сорта картофеля охарактеризованы оригинаторами и Госсортокомиссией по устойчивости только к четырем возбудителям: раку (все устойчивы), золотистой картофельной нематоде (устойчивы 15 сортов) и морщинистой и полосчатой мозаике, возбудителем которых является PVY (устойчивы 8 сортов). Между тем ни отечественные, ни зарубежные сорта в Госреестре не охарактеризованы на устойчивость к таким вредоносным заболеваниям, как фитофтороз, ризоктониоз, обыкновенная и серебристая парша, антракноз, а также к другим вирусным болезням. Информацию по устойчивости некоторых новых отечественных сортов можно найти в издании «Сорта картофеля российской селекции» (2018).

На сайтах зарубежных селекционных фирм приводятся характеристики устойчивости к наиболее экономически значимым болезням, в частности к цистообразующим нематодам, вирусу картофеля Y (PVY) и к фитофторозу. Однако возможны расхождения в оценках, сделанных за рубежом и в условиях разных агроклиматических зон России. Связано это прежде всего с различным составом популяций патогенов и особенностями экологических условий. В этом плане большое значение имеет информация о генах устойчивости сортов, особенно тех, что обладают устойчивостью к различным патотипам и видам цистообразующих нематод, поскольку фитопатологические тесты длительны, трудоемки и не всегда возможны для карантинных объектов. В зарубежных селекционных центрах давно и успешно используются методы ДНКмаркирования для оценки генетической защищенности сортового генофонда. В последнее время такие данные появляются и для отечественных сортов. Так, сотрудниками ВИР проведен скрининг 225 отечественных сортов картофеля, 114 из которых входят в «Государственный реестр селекционных достижений, допущенных к использованию» (Антонова и др., 2016; Клименко и др., 2017; Гавриленко и др., 2018). Наиболее эффективный маркер 57R гена Н1 выявлен лишь у 28 % изученных сортов (и у 26 %, входящих в Госреестр); эти сорта по данным госсортоиспытаний являются устойчивыми к патотипу Ro1 золотистой картофельной нематоды.

В связи с экономической значимостью особо опасных болезней, характеристика поражаемости современных сортов возбудителями этих болезней имеет важное значение для селекции и семеноводства, а также для выбора сортов для возделывания, особенно семенного картофеля, в эпидемиологически опасных зонах. Целью работы была оценка устойчивости к возбудителям фитофтороза и глободероза и определение поражаемости возделываемых сортов картофеля грибными и оомицетными болезнями в различных агроклиматических зонах России. 

## Материалы и методы

Растительный материал. Экспериментальной выборкой для изучения устойчивости к патогенам послужили 42 сорта зарубежной и 1 сорт (восприимчивый контроль) отечественной селекции. Для 21 сорта из выборки была проведена фитопатологическая экспертиза образцов семенного картофеля из различных регионов РФ.

Оценка на устойчивость к ЗКН. Оценку на устойчивость сеянцев картофеля к G. rostochiensis проводили по методике, рекомендованной Европейской и Средиземноморской организацией по защите растений, с небольшими модификациями (OEPP/EPPO, 2006). Исследуемые сорта высаживали в пластиковые горшки объемом 500 см^3^, наполовину наполненные почвой (по одному клубню в каждый горшок). В качестве инфекционного материала для инокуляции сортов использовали популяцию золотистой картофельной нематоды, отобранную в Ленинградской области из известного очага G.rostochiensis и типированную до патотипа Ro1 (Limantceva et al., 2014).

В каждый горшок вносили инокулюм ЗКН из расчета 1500 яиц и личинок в 100 см^3^ почвы. Яйца и личинки получали методом раздавливания цист ЗКН в капле воды на предметном стекле. После инокуляции клубней дополнительно досыпали почву до верха горшка. Горшки оставляли в контролируемых условиях при температуре 22 °С. В качестве восприимчивого контроля использовали сорт Невский, в качестве устойчивого контроля – сорт Red Scarlett. Сорта высаживали в десятикратной повторности и двукратной аналитической. Учет результатов заражения проводили через три месяца, что является достаточным промежутком времени для развития цист ЗКН. Экстракцию цист из почвы осуществляли методом флотации (Turner, 1998). Экстрагированные цисты переносили на покровные стекла в каплю воды, раздавливали и подсчитывали количество яиц и личинок в них

Оценку результатов заражения проводили по шкале с подразделением образцов на группы: балл 9 (относительная восприимчивость <1 %) – Very high; балл 8 (1.1–3 %) – High/very high; балл 7 (3.1–5 %) – High; балл 6 (5.1–10 %) – Moderate/high; балл 5 (10.1–15 %) – Moderate; балл 4 (15.1–25 %) – Moderate/low; балл 3 (25.1–50 %) – Low; балл 2 (50.1–100 %) – Low/very low; балл 1 (>100 %) – Very low. К классу устойчивых (R) относили растения, тип реакции которых соответствовал 7–9 баллам, среднеустойчивых (RS) – 4–6 баллам, восприимчивых (S) – 1–3 баллам. Относительную восприимчивость определяли по формуле: количество яиц и личинок исследуемого образца делили на количество яиц и личинок эталонного восприимчивого сорта и умножали на 100 %.

Оценка на устойчивость к фитофторозу. Лабораторный скрининг сортов картофеля на устойчивость к фитофторозу проводили по стандартной методике (Brylińska, Śliwka, 2017). В качестве инфекционного материала использовали изолят VZR17, включающий все гены вирулентности 1.2.3.4.5.6.7.8.9.10.11.

Отделенные листья помещали в поддоны (45×35 см) на влажную фильтровальную бумагу, абаксиальной стороной вниз: по 3 листа каждого образца, по 3 листа восприимчивого сорта Bintje и по 3 листа устойчивого контроля сорта Sarpo Mira, в двукратной биологической повторности. Для заражения использовали инокулюм, выдержанный в течение 30 мин при температуре 10–12 °С для стимуляции выхода зооспор. Инфекционная нагрузка составляла 50 000 спорангиев/мл. Инокулюм наносили по одной капле по центру каждого листа между центральной и отходящей жилками. Объем капли составлял 30 мкл. Инокулированные листья выдерживали в течение 24 ч в темноте при температуре 16 °С. На протяжении всего эксперимента поддоны были закрыты стеклянными крышками для поддержания постоянной влажности (80–100 %). Через сутки после инокуляции листья переворачивали абаксиальной стороной вверх, после чего кюветы переносили в климатический бокс с температурой 16 °С, интенсивностью освещения 1600 лк и 16-часовым фотопериодом.

Учет результатов заражения проводили на 6-е сутки после инокуляции, по стандартной шкале с подразделением образцов на группы: балл 9 (0 % пораженной площади) – Very high; балл 8 (3 %) – High/very high; балл 7 (3.1– 10 %) – High; балл 6 (10.1–25 %) – Moderate/high; балл 5 (25.1–75 %) – Moderate; балл 4 (75.1–90 %) – Moderate/low; балл 3 (90.1–97 %) – Low; балл 2 (97.1–99 %) – Low/ very low; балл 1 (100 %) – Very low. Растения с типом реакции, соответствующим баллам 7–9, относили к классу устойчивых (R), 4–6 – среднеустойчивых (RS), 1–4 – восприимчивых (S).

Молекулярный скрининг. ДНК выделяли из листьев тепличных растений методом модифицированной СТАВэкстракции (Gavrilenko et al., 2013). Скрининг проводили на наличие маркеров гена Н1, контролирующего устойчивость к патотипам Ro1 и Ro4 G. rostochiensis (Dalamu et al., 2012). Для маркеров гена Н1 показана различная эффективность в молекулярном скрининге. Высокий уровень специфичности демонстрировал SCARмаркер 57R и низкую эффективность – CAPS-маркер этого гена, 239E4left/AluI (Антонова и др., 2016). В то же время в исследованиях зарубежных коллег сообщалось о высокой частоте встречаемости CAPS-маркера 239E4left/ AluI гена Н1 у нематодоустойчивых зарубежных сортов. Поэтому в данной работе использовали оба эти маркера. Праймеры для работы подбирали по литературным источникам (табл. 1). Использовали SCAR-маркер 57R, интегрированный в ассоциированную с устойчивостью область ‘341 Kb’ локуса Н1, и CAPS-маркер 239E4left/ AluI, расположенный на расстоянии 2.1 сМ от ассоциированного с устойчивостью локуса (Finkers-Tomczak et al., 2011).

**Table 1.**
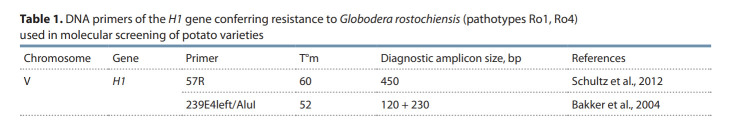
DNA primers of the H1 gene conferring resistance to Globodera rostochiensis (pathotypes Ro1, Ro4) used in molecular screening of potato varieties

ПЦР проводили в 20 мкл реакционной смеси состава: 40 нг тотальной ДНК, 1×реакционный буфер, 2.5 мМ MgCl2, 0.4 мМ каждого из dNTP, по 0.25 мкМ прямого и обратного праймера и 1 ед. Taq-полимеразы («Диалат», Москва). Условия реакции соответствовали указанным в литературе.

В качестве положительных контролей для маркера 57R использовали сорта Живица, Сударыня и Sante, для которых наличие диагностического фрагмента было установлено нами ранее; контролем для маркера 239E4left/AluI служил сорт Sante (Антонова и др., 2016). Рестрикцию осуществляли ферментом AluI («СибЭнзим»), используя протокол фирмы-изготовителя. Фрагменты ДНК разделяли электрофорезом в 2 % агарозных гелях с окрашиванием бромистым этидием и визуализацией в УФ-свете.

Фитопатологический анализ. Отбор клубневых проб и диагностику осуществляли в соответствии с методиками, приведенными в ГОСТ 33996-2016 «Картофель семенной. Технические условия и методы определения качества» (2017), и международным стандартом по семенному картофелю UNECE S-1 (2018). Для каждого анализируемого образца отбиралось по 10 точечных проб, составлявших в совокупности не менее 250 клубней. Однако для образцов, датированных 2018 г., количество анализируемых клубней варьировало от 20 до 100 шт.

Экспериментальная выборка при фитопатологической экспертизе семенного картофеля состояла из 92 образцов 39 сортов из четырех федеральных округов РФ: 24 образца из Приволжского, 35 – из Северо-Западного, 8 – из Центрального, 9 – из Северо-Кавказского.

Диагностику оомицетных, грибных и бактериальных болезней выполняли, руководствуясь информацией, представленной в специализированных компендиумах (Compendium…, 1981, 2001; Diseases…, 2008), а также с использованием определителей UNECE (2014) и AHDB (2018).

Анализировали каждый клубень изучаемого образца. Клубни с нетипичной симптоматикой или малозаметными патологическими изменениями помещали во влажные камеры. Поверхность клубней предварительно дезинфицировали 70 % этиловым спиртом с последующей промывкой дистиллированной водой. При необходимости клубни разрезали на небольшие ломтики размером не менее 1 см. Инкубационный период составлял от 3 до 14 дней, в зависимости от возбудителя, при постоянной температуре 20 °С и 100 % влажности.

Выделение в чистую культуру возбудителей болезней осуществляли с использованием картофельной (картофель 200 г, агар-агар 20 г, Н2О 1000 мл) и ржаной (рожь 60 г, сахароза 20 г, агар-агар 15 г, Н2О 1000 мл) сред.

## Результаты

**Устойчивость сортов картофеля к золотистой картофельной нематоде**

Согласно полученным данным, из 41 сорта зарубежной селекции только три сорта (или 7.3 % от всех изученных) оказались восприимчивыми к ЗКН: Bintje, Desiree и Sarpo Mira; остальные проявили себя как устойчивые. Промежуточных групп устойчивости не было выявлено (табл. 2). 

**Table 2.**
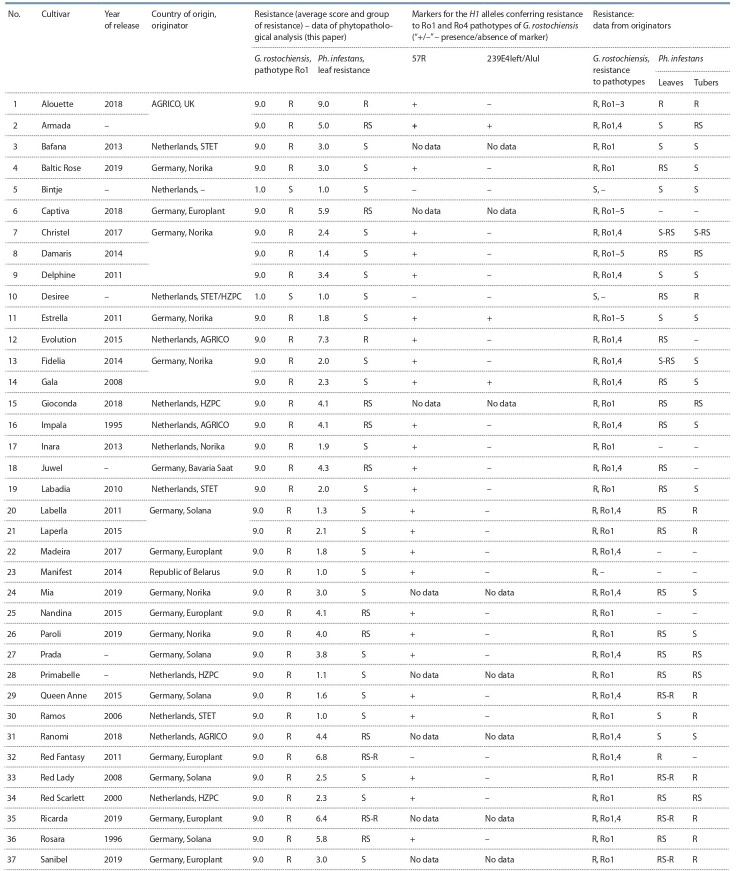
Characteristics of resistance of modern potato cultivars to G. rostochiensis and Ph. infestans Note: Categories of sustainability are based on the data provided on the Internet portals of originators, the State Register of Breeding Achievements (2019), and catalogs. Designations: R, resistant; RS, moderately resistant; S, susceptible; –, no data.

Среди 29 изученных нематодоустойчивых сортов частота встречаемости генотипов со SCAR-маркером 57R гена Н1, определяющего устойчивость к патотипу Ro1 G. rostochiensis, очень высокая – 96.6 % (28 из 29 устойчивых сортов). Исключением является устойчивый (по данным фитопатологических тестов) сорт Red Fantasy, у которого не выявлены маркеры гена Н1 (рис. 1, см. табл. 2).

**Fig. 1.**
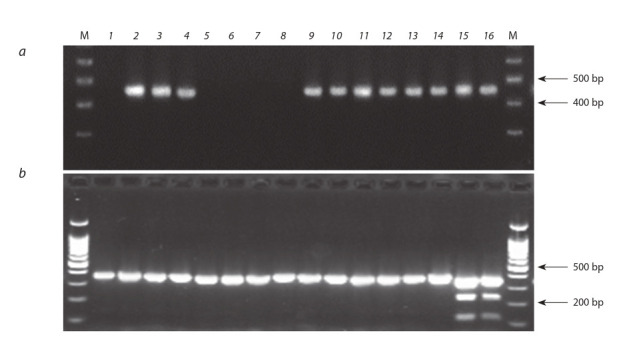
Molecular screening of potato cultivars using DNA markers 57R (a) and 239E4left (b) for the H1 gene. Varieties: 1. Nevskiy, 2. Alouette, 3. Baltic Rose, 4. Nandina, 5. Sarpo Mira, 6. Bintje, 7. Desiree, 8. Red Fantasy, 9. Christel, 10. Madeira, 11. Ramos, 12. Queen Anne, 13. Delphine, 14. Labella, 15. Armada, 16. Estrella. M, molecular ladder 100 bp+1.5 Kb (SibEnzyme, Russia).

Коэффициент корреляции Пирсона между наличием маркера 57R и данными об устойчивости сортов к патотипу Ro1 составил +0.88 (n = 33, вместе с контролем – сорт Невский). Другой маркер гена Н1 – CAPS-маркер 239E4Left/AluI – обнаруживался гораздо реже. Этот маркер наряду с 57R был детектирован только у трех сортов (Armada, Estrella, Gala), все они проявляли устойчивость к ЗКН (см. табл. 2).

Устойчивость сортов картофеля к возбудителю фитофтороза 

Только два сорта зарубежной селекции оказались абсолютно устойчивыми к возбудителю фитофтороза картофеля: Alouette и Sarpo Mira (см. табл. 2). Остальные 39 сортов показали разный уровень устойчивости или восприимчивости. Из них можно отметить три сорта: Evolution, Red Fantasy и Ricarda, которые выделялись достаточно высоким уровнем устойчивости (средний балл 7.3, 6.8 и 6.4 соответственно). 

Оценка фитосанитарного состояния семенного картофеля различных сортов в регионах РФ

Результаты фитопатологического анализа партий элитного и репродукционного семенного картофеля свидетельствуют, что во всех регионах на семенном картофеле выявлены: Ph. infestans, R. solani, H. solani, Streptomycesspp., P. pustulans, С. coccodes, Fusarium spp., Phoma spp. (табл. 3). Бугорчатая парша P. pustulans отмечена повсеместно, кроме Северо-Кавказского федерального округа. Порошистая парша S. subterranea f. sp. subterranea отмечена только в одном образце сорта Вектор в Приволжском федеральном округе

**Table 3.**
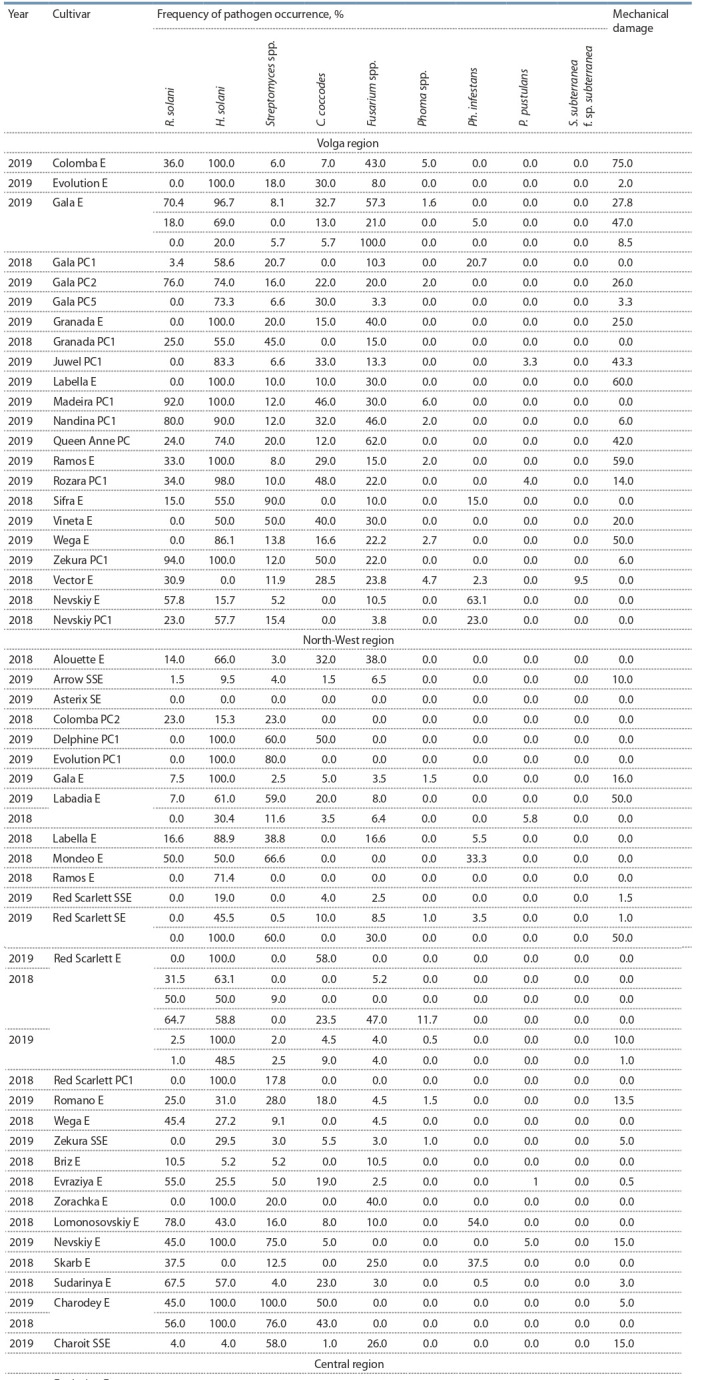
Phytopathological analysis of seed potato from various regions of the Russian Federation Note: Elite seed potatoes: SSE – super-super elite, SE – super-elite, E – elite. Seed potato: PC1 – the first reproduction, PC2 – the second reproduction, etc.

Наибольшее распространение на всех сортах получили ризоктониоз и серебристая парша (рис. 2). Стопроцентное поражение клубней серебристой паршой отмечено в различных регионах на сортах Red Scarlett (СЭ, Э и РС1-2), Evolution (Э, РС1), Labella (Э), Colomba (Э), Gala (Э), Невский (Э). В Северо-Кавказском ФО серебристая парша была отмечена только на 6.0 % образцов сорта Невский (СЭ). Широкое распространение получил антракноз картофеля. Сильнее всего были поражены клубни сорта Red Scarlett (Э и РС2) – от 50 до 71.4 % в Центральном федеральном округе. Максимальное поражение фитофторозом отмечено на сорте Невский (Э) – 63.1 %.

## Обсуждение

Одним из приоритетных направлений селекции является создание сортов, сочетающих в себе групповую устойчивость, в первую очередь к таким опасным заболеваниям, как фитофтороз, ризоктониоз, комплекс паршей, вирусные, бактериальные и нематодные болезни. В «Государственном реестре селекционных достижений, допущенных к использованию» на территории Российской Федерации (2019), данные по устойчивости сортов картофеля к комплексу заболеваний отсутствуют. Среди обязательных характеристик приведены показатели устойчивости к раку, золотистой картофельной нематоде и вирусуY. Для некоторых сортов указана их устойчивость/ восприимчивость к фитофторозу. Наши результаты свидетельствуют, что патогенный комплекс на современных сортах картофеля значительно шире и может быть определяющим для конкурентоспособности сорта на отечественном рынке сортов. 

Для сортов зарубежной селекции на англоязычных интернет-ресурсах в полной мере отражены все сортовые особенности, включая устойчивость к основным заболеваниям по 9-балльной шкале. Стоит отметить, что для большинства сортов приведена информация по устойчивости к фитофторозу ботвы и клубней и к вирусу картофеля Y (PVY). Наличие остальной информации по устойчивости/восприимчивости к болезням варьирует в зависимости от семеноводческой компании и страны-производителя. Общеизвестно, что оценка сорта на устойчивость к заболеваниям проводится на территории страны-оригинатора к местным популяциям или расам патогенов, которые могут значительно отличаться от представленных в России. Например, при фитопатологическом анализе сорта Sifra, который по данным оригинатора высокоустойчив к фитофторозу по клубням (8 баллов, где 9 – абсолютная устойчивость) (см. табл. 3), количество пораженных клубней в Приволжском федеральном округе составило 15 %. У сорта Labella, который тоже высокоустойчив к фитофторозу по клубням, в Северо-Западном федеральном округе выявлено 5.5 % пораженных клубней, а в Северо-Кавказском – 25.0 %.

Такая же ситуация наблюдается и по вирусу картофеля Y. По данным оригинатора, сорт Alouette является иммунным к этому виду вируса, но нами было выявлено три пораженных клубня из четырех (Yanagisawa et al., 2019). Клубни высокоустойчивых к вирусуY сортов Queen Anne, Rozara и Adretta, районированных в Дальневосточном федеральном округе, были поражены этим вирусом с частотой 1 из 22, 1 из 4 и 3 из 4 соответственно. Сорт Red Lady, по сведениям оригинатора, среднеустойчив к вирусу Y и высокоустойчив к штамму PVY^NTN^, однако все 29 клубней (100 %) этого сорта были поражены штаммами PVY^NTN^(A), PVY^NTN^(B), PVY^N^W(A), PVY^N^W(В) (Yanagisawa et al., 2019).

Полученные данные по устойчивости сортов к ЗКН полностью коррелируют с информацией, приведенной фирмами-производителями и в Государственном реестре селекционных достижений (2019). Все изученные современные зарубежные сорта картофеля, кроме трех (Bintje, Desiree и Sarpo Mira, которые часто используются в качестве контроля восприимчивости), являются полностью иммунными к патотипу Ro1 ЗКН. Остальные зарубежные сорта картофеля, включенные в Госреестр и разрешенные к выращиванию на территории РФ, также отличаются высокой устойчивостью к этому патотипу ЗКН.

По данным Госреестра селекционных достижений, 254 из 455 сортов картофеля устойчивы к ЗКН (рис. 3). Тенденция превалирования устойчивых сортов над восприимчивыми наблюдается начиная с 2013 г. Однако это связано с включением в Госреестр многочисленных иностранных сортов, которые высокоустойчивы не только к Ro1 патотипу ЗКН, но и к другим, включая групповую устойчивость к бледной картофельной нематоде (например, сорта Laperla и Prada, фирма-оригинатор Solana). Свыше половины (124 из 216, или 57.4 %) отечественных сортов селекции РФ и стран СНГ, включенных в Госреестр, к сожалению, по большей части являются восприимчивыми, несмотря на то что признак нематодоустойчивости считается одним из важнейших при создании новых сортов картофеля.

**Fig. 2.**
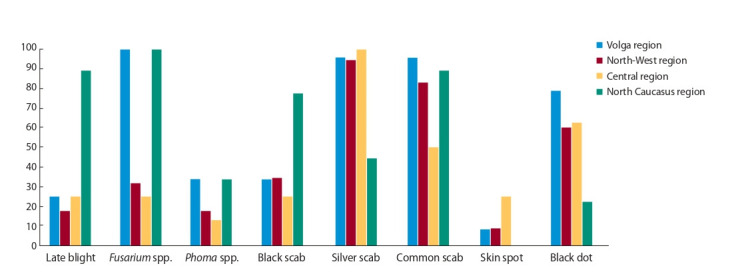
The prevalence of fungal and oomycete diseases on seed potato in various agroclimatic zones of Russia (average for 92 samples).

**Fig. 3.**
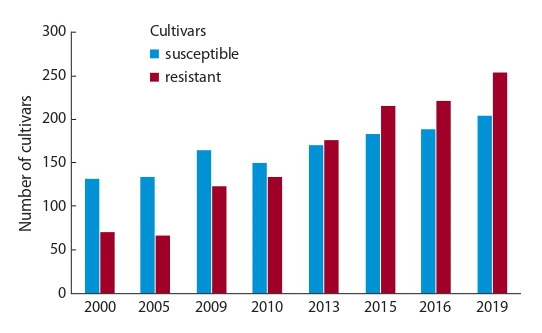
The numbers of potato cultivars resistant and susceptible to Globodera rostochiensis (Ro1 patotype) released from 2000 to 2019.

Устойчивость к ЗКН является моногенной и обусловлена наличием генов H1 или Gro1–4 устойчивости, локализованных на хромосомах V и VII соответственно. Нематодоустойчивые сорта, созданные селекционерами разных стран, чаще всего несут ген Н1 (Shultz et al., 2012). Подобная закономерность выявлена и для отечественных нематодоустойчивых сортов, 98 % которых обладали маркерами гена Н1 и только 2 % – маркерами гена Gro1–4 (Клименко и др., 2017). Зарубежные сорта картофеля активно используются отечественными селекционерами в программах по выведению новых сортов; приведенная авторами (Клименко и др., 2017) информация об устойчивости зарубежных сортов, а также о наличии маркера 57R гена Н1 важна при подборе пар для скрещиваний и в программах по пирамидированию генов устойчивости, особенно к карантинным объектам.

Сорта Alouette и Sarpo Mira, по данным оригинаторов, являются иммунными к фитофторозу и в наших экспериментах подтвердили этот статус. Устойчивость сорта Alouette обусловлена наличием генов Rpi-R3a, Rpi-R3b, Rpi-vnt1 (Armstrong et al., 2019), а у сорта Sarpo Mira детерминирована генами R3a, R3b, R4, R8, Rpi-Smira1 и Rpi-Smira2 (Rietman et al., 2012). Сорт Evolution считается среднеустойчивым (RS), однако в проведенном эксперименте был оценен как устойчивый (балл 7.3).

Сорта Baltic Rose, Damaris, Desiree, Gala, Labella, Laperla, Mia, Sanibel, Zekura и 7 for 7 отнесены нами к восприимчивым, хотя в характеристиках оригинаторов указывалась средняя устойчивость к фитофторозу. Восприимчивыми оказались и сорта Queen Anne и Red Lady, которые по данным оригинатора имеют устойчивость выше средней. При проведении фитопатологического анализа семенного картофеля пораженные фитофторозом клубни были обнаружены у сортов Desiree, Labella, Queen Anne и Red Fantasy, которые по данным оригинаторов считаются устойчивыми к клубневой форме фитофтороза

По групповой устойчивости к обоим возбудителям, золотистой картофельной нематоде и фитофторозу, выделились четыре районированных зарубежных сорта: Alouette, Evolution, Red Fantasy, Ricarda.

Результаты наших исследований свидетельствуют, что распространенность болезней на картофеле варьировала в зависимости от сорта, репродукции семенного материала и зоны выращивания. Зональные различия по распространенности болезней на семенном картофеле отчетливо проявились только по Северо-Кавказскому ФО. В отличие от других регионов РФ, в Северо-Кавказском ФО отсутствовало поражение картофеля антракнозом и только в одном образце сорта Невский (СЭ) обнаружено поражение серебристой паршой.

Повсеместное сильное поражение клубней ризоктониозом выявлено во всех исследованных регионах. Максимальное распространение болезнь получила на сортах Gala (70.4 %), Red Scarlett (64.7 %), Невский (57.8 %), относящихся к категории «элита».

Серебристая парша отмечена в различных регионах на всех изученных сортах картофеля, кроме некоторых из Северо-Кавказского ФО. Вредоносность этого заболевания состоит в поражении глазков, в результате чего клубни теряют всхожесть или дают ослабленные побеги, а также в поражении корневой системы и столонов, вследствие чего образуются невыравненные по размеру клубни, часто уродливые. Пораженные клубни чаще подвергаются развитию вторичной инфекции – фитофтороза и грибов Fusarium spp.

Неожиданно сильное распространение получил антракноз картофеля. Сильнее всего были поражены клубни сорта Red Scarlett (Э и РС2) (50.0–71.4 %) в Центральном федеральном округе. Устойчивость к антракнозу никогда не входила в параметры хозяйственно ценных признаков сортов картофеля. Это заболевание отсутствует в актуальном ГОСТ 33996-2016, что отчасти и способствует его распространению. Сильное поражение вегетирующих растений картофеля антракнозом отмечено нами в Северо-Западном и Дальневосточном федеральных округах на сортах Labadia, Labella, Невский и др. Вредоносность болезни состоит в преждевременном отмирании ботвы и гниении клубней во время вегетации и хранения. При диагностике основным признаком является наличие склероциального уплотнения мицелия под кожурой клубня, с выходом на поверхность и образованием щетинок. Широкое распространение антракноза и серебристой парши и очевидная их вредоносность стали новым вызовом для селекции картофеля.

Все проанализированные образцы были поражены комплексом грибов Fusarium spp. На сорте Gala их распространенность достигала 100 % в Приволжском ФО. Отчасти это связано с тем, что именно в этом округе зафиксировано наибольшее количество механически поврежденных клубней (от 2 до 75 % в зависимости от образца), что, по нашему мнению, способствовало такому сильному распространению сухой фузариозной гнили.

ГОСТ 33996-2016 устанавливает жесткие нормативные требования, предъявляемые к категориям картофеля по пораженности сухими гнилями, в частности фузариозом: 0.5 % для категорий элитного и 1.0 % для репродукционного семенного картофеля. Ни один проанализированный образец, вне зависимости от репродукции, не соответствовал предъявляемым требованиям, что свидетельствует о неудовлетворительном фитосанитарном состоянии семенного картофеля. Отсутствие поражения фузариозной инфекцией таких сортов, как Mondeo, Asterix, Delphine, Чародей, Зорачка, не может быть доказательством устойчивости к патогену, необходимо их дальнейшее изучение. 

Возбудитель фитофтороза картофеля был выявлен во всех федеральных округах, причем наибольшее распространение на районированных сортах получил в Северо-Кавказском ФО (88.8 %). С 2018 г. фитофтороз картофеля отсутствует в регламенте контроля при проведении сертификации семенного материала по новому ГОСТ 33996-2016 (2017), поэтому в настоящее время отсутствуют допустимые критерии по пораженности клубней семенного картофеля. Однако, согласно ЭПВ (Алехин и др., 2016), не допускается присутствия пораженных клубней в семенном материале. Не выявлено корреляционной зависимости по поражению различных категорий сортов картофеля заболеваниями, скорее наоборот: именно элитный семенной картофель был поражен болезнями сильнее, чем репродукционный (см. табл. 3).

Наиболее представленными во всех федеральных округах оказались сорта картофеля, включенные в Госреестр (2019) много лет назад: Невский (год включения 1982), Red Scarlett (2000) и Gala (2008), менее представленными – сорта Evolution (2015), Colomba (2013) и Labella (2010). Все перечисленные сорта районированы в Северо-Западном ФО, остальные сорта присутствовали в трех регионах в разных соотношениях (см. табл. 3).

## Заключение

Все зарубежные сорта картофеля, внесенные в Госреестр селекционных достижений, отличаются высокой устойчивостью к распространенному на территории Российской Федерации патотипу Ro1 G. rostochiensis. Часть из них генетически защищена и против других патотипов ЗКН. Это косвенно свидетельствует об эффективном использовании молекулярных маркеров генов устойчивости, так как фитопатологические тесты длительные и трудоемкие и могут быть проведены только в контролируемых условиях карантинных лабораторий. У 96.6 % изученных нематодоустойчивых сортов выявлен маркер гена Н1 устойчивости к патотипу Ro1 G. rostochiensis, восприимчивые сорта этим маркером не обладали. Подтверждена высокая устойчивость сортов зарубежной селекции Alouette и Sarpo Mira к возбудителю фитофтороза. Обнаружены расхождения в характеристике устойчивости сортов картофеля к фитофторозу, представленной зарубежными оригинаторами и полученной нами при оценке устойчивости и фитопатологической экспертизе семенного картофеля, возделываемого на территории РФ. Сорт Alouette компании Agrico, UK отличается групповой устойчивостью к ЗКН и фитофторозу. 

Во всех регионах изучения на семенном картофеле выявлен комплекс оомицетных и грибных болезней, среди которых преобладали ризоктониоз, сухая фузариозная гниль и серебристая парша. Отмечено неожиданно широкое распространение антракноза картофеля. По-видимому, назрела необходимость определить наличие устойчивости к этим болезням как важный хозяйственно ценный признак и направить усилия фитопатологов и селекционеров на создание генетически охарактеризованного исходного материала для селекции на устойчивость. Большая часть районированных сортов не удовлетворяет даже минимальным требованиям, предъявляемым к семенному материалу. Практически весь семенной материал не соответствует требованиям ГОСТ и ЭПВ, что свидетельствует о нарушении технологии возделывания и защиты. 

## Conflict of interest

The authors declare no conflict of interest.
